# Chondrosarcoma of the nasal cavity in a patient with Maffucci syndrome: case report and review of the literature

**DOI:** 10.1186/1477-7819-12-387

**Published:** 2014-12-17

**Authors:** Teresa B Steinbichler, Florian Kral, Susanne Reinold, Herbert Riechelmann

**Affiliations:** Department of Otorhinolaryngology, Medical University of Innsbruck, Anichstrasse 35, A- 6020 Innsbruck, Austria; Department of Pathology, Medical University of Innsbruck, Müllerstrasse 44, A-6020 Innsbruck, Austria

**Keywords:** Chondrosarcoma, Enchondroma, Hemangioma, Maffucci syndrome

## Abstract

**Introduction:**

Maffucci syndrome is a rare, congenital, non-hereditary mesodermal dysplasia, manifested by multiple enchondromas and hemangiomas. Malignant transformation of these lesions is seen in up to 40% of the cases.

**Case report:**

We present a case of a patient with Maffucci syndrome and an associated chondrosarcoma of the nose. Treatment consisted of surgical resection. Because of the low grade of the tumor, additional treatment, such as radiotherapy, was not necessary.

**Conclusion:**

Maffucci syndrome is an exceedingly rare mesodermal dysplasia. Its manifestation in the head and neck region is even less common. Malignant transformation of the associated enchondromas is common, and should be considered whenever a change of the clinical course occurs. Random, periodically performed X-ray examinations give little additional information on malignant transformation and are considered useless.

## Background

Maffucci syndrome is a rare, non-hereditary mesodermal dysplasia. It is characterized by multiple enchondromas and hemangiomas, especially in the extremities [1]. A manifestation in the head and neck region is rare. There are only two case reports that describe a primary involvement of the nasal cavities and paranasal sinuses [2, 3].

The enchondromas usually develop near to growth plate cartilage and may result from deregulated proliferation and differentiation of chondrocytes during physiological enchondral ossification [4]. Complications include spontaneous fracture, resulting in skeletal deformity and the possibility of malignant transformation in up to 40% of the cases [1].

Three types of vascular lesions are associated with Maffucci syndrome: cavernous hemangiomas, phlebectasias and lymphangiectasia-lymphangiomas [5].

The syndrome can be associated with benign and malignant tumors, like parathyroid and pituitary adenoma, astrocytoma and breast cancer [5].

The disease occurs usually in one side of the body. The bone and vascular lesions develop at birth or in early childhood and may be progressive [5, 6]. The average age at diagnosis is 12 years and diagnosis is made on clinical grounds [1].

Surgical intervention is not required in the absence of complications [7].

Cartilaginous tumors of the head and neck are rare. The most frequent sites are the larynx and spheno-ethmoidal region. Typical clinical symptoms of cartilaginous nasal tumors include nasal mass, obstruction, headache and epistaxis. The therapy is wide surgical excision [8].

## Case presentation

A 39-year-old white woman presented to the Department of Otorhinolaryngology-Head and Neck surgery, Medical University of Innsbruck, with an 18-month history of nasal obstruction. One year ago, an ambulatory ear, nose and throat examination and tissue biopsy had revealed a chondroma of the left nasal cavity. Surgical resection at that time was not possible because of gravidity. The patient was now presenting to our clinic because of a worsening of symptoms during the last few weeks.

**Figure 1:**
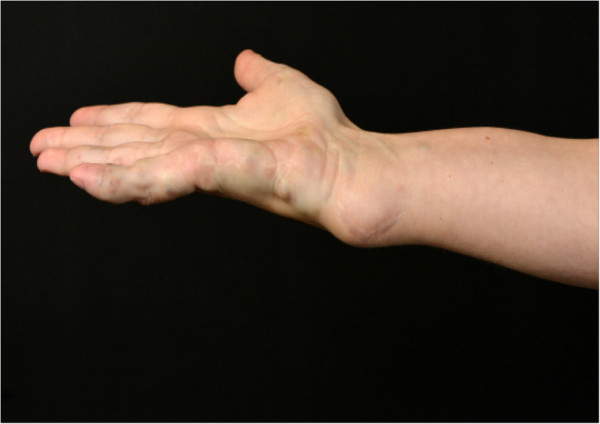
**Right wrist with a large hemangioma and a few smaller ones on the metacarpophalangeal joint, and multiple scars after resection of relapsing hemangiomas.**

Her medical history included Maffucci syndrome, diagnosed in early childhood. Relapsing hemangiomas, especially in the metacarpophalangeal joints of the right hand and chondromas in the left lower extremity had necessitated prior surgical interventions (Figure 1).

The initial endoscopic evaluation revealed a large mass in the left nasal cavity with no signs of inflammation or destruction of surrounding tissue.

**Figure 2:**
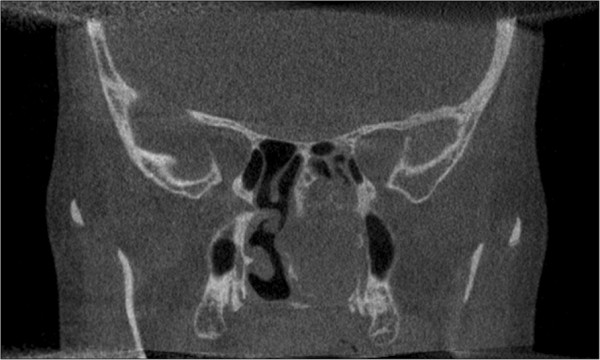
**Coronal non-contrasted computed tomography scan of the skull, showing a tumorous lesion in the left nasal cavity extending 3.7 × 3.2 × 2.6 cm in diameter with partial infiltration of the surrounding structures.**

**Figure 3:**
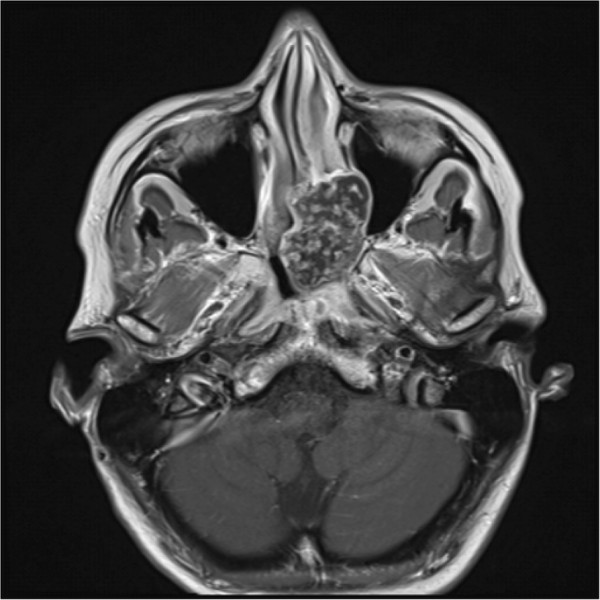
**Axial T1-weighted magnetic resonance imaging scan with gadolinium of the skull showing the tumorous lesion in the left nasal cavity with infiltration of the surrounding structures.**

Computed tomography confirmed the clinical findings and showed a tumorous mass of diameter 3.7 × 3.2 × 2.6 cm affecting the nasal cavity, the posterior naris, the base of the sphenoid sinus and the lateral wall of the maxillary sinus (Figure 2). Magnetic resonance imaging of her skull revealed an infiltration of the nasal septum and the base of the sphenoid sinus (Figure 3).

A complete surgical resection of the tumor was decided. The tumor was resected via a transnasal endoscopic approach with the help of computer-assisted navigation. During surgery, the smooth and lobulated tumor was found to have infiltrated the nasal septum and the anterior wall of the sphenoid sinus. Consequently the posterior part of the nasal septum and the anterior wall of the sphenoid sinus had to be removed, resulting in a permanent connection of both nasal cavities. The tumor was affecting the left maxillary sinus but without infiltration of the surrounding bone, so it could easily be removed. All surrounding bony structures were smoothed with a sinus drill device. Frozen sections were taken from the margins and confirmed complete tumor resection.

Possible surgical complications could be divided into intracranial, orbital and local complications. Local complications include anosmia, bleeding and septal abscess. Intracranial complications include rhinoliquorrhoea leading to meningitis, encephalitis or intracranial abscesses. Possible orbital complications include double vision resulting from motility dysfunction, loss of vision, and orbital infections or intraorbital hematoma. Postoperatively, none of these complications was noted.

**Figure 4:**
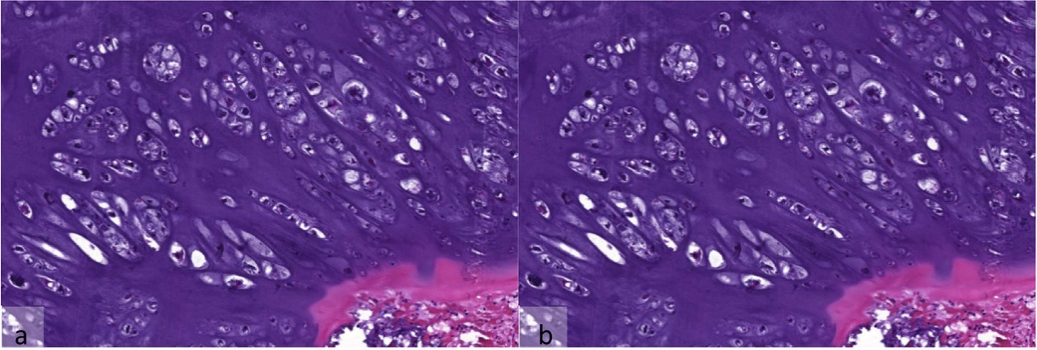
**Microscopical features of the pathological sections of tumor.** Hyaline cartilage with an increased cell densitiy and cellular atypia with irregular and hypochromatic nuclei. **(a)** Hematoxylin-eosin staining, ×2. **(b)** Hematoxylin-eosin staining, ×10.

A histological workup revealed a mesenchymal tumor with a few enlarged chondrocytes with irregular and hypochromatic nuclei within a hyaline cartilaginous matrix. The cell density was increased but mitotic figures were rare and had a bland histological appearance so that the tumor was classified as a conventional low-grade chrondrosarcoma (Figure 4).

Because of the complete surgical resection of the chondrosarcoma, no additional radiotherapy was necessary. The interdisciplinary head and neck tumor board decided on a clinical follow-up. Because of the recurrent hemangiomas, therapy with propranolol (Inderal; Astra Zeneca Inc., Vienna, Austria) was initiated.

Chondrosarcomas are the third most common type of primary bone tumors (after osteosarcoma and myeloma) [9]. Only 10% are found in the head and neck region [10]. According to our histological findings the majority of chondrosarcomas of the skull base are conventional chondrosarcomas with good or moderate differentiation. Other rare types that present at the skull base are poor differentiated mesenchymal chondrosarcomas or extraskeletal myxoid chondrosarcoma [11].

The mean age at diagnosis varies between 30 and 60 years [9]. Presenting symptoms are nasal mass and obstruction and depend on the involvement of adjacent structures [12]. The main affected sites are the sphenoid and maxillary sinus [13]. Overall survival after 5 years is estimated at 80.7% [14], depending on size, localization and grade [15]. The metastasis rate is higher in primary and dedifferentiated chondrosarcomas than in secondary ones [16].

Surgical resection is the most effective therapy. The resection margins should be determined in relation to the grade of differentiation of the tumor. While wide bloc excision is recommended for high-grade chondrosarcomas, local curettage is sufficient for low-grade tumors. Because of the low percentage of dividing cells, poor vascularization and the production of extracellular matrix, chondrosarcomas are relatively radio- and chemotherapy resistant. Radiotherapy is feasible after incomplete resection or whenever resection is not possible, to achieve local control [9].

Patients with Maffucci syndrome have a 25% to 30% risk of developing chondrosarcomas and are usually younger than those with primary chondrosarcoma [15]. The chondrosarcomas usually present at the upper and lower extremities. An involvement of the head and neck region in Maffucci syndrome is exceedingly rare [6]. Enchondromas of the axial skeleton have a higher risk of malignant transformation than those found in the smaller bones of the extremities. Enchondromas in patients with Maffucci syndrome have a more locally aggressive growth pattern, so that the differential diagnosis towards a low-grade chondrosarcoma is even more difficult than usual [1].

Random, periodically performed X-ray examinations give little additional information on malignant transformation and are considered useless. Changes in the clinical symptoms should be an indication for surgical intervention rather than radiological findings [1]. The clinical symptoms our patient presented with determined the immediate surgical intervention in this case. Additional therapy was not necessary because of the low grade of the lesion, for which regional curettage is sufficient [9].

## Conclusion

Maffucci syndrome is an exceedingly rare mesodermal dysplasia. Malignant transformation of the associated enchondromas is common, and should be considered whenever a change of the clinical course occurs. If complete surgical resection can be achieved, no additional treatment like radiotherapy is necessary. The prognosis of these chondrosarcomas is quite favorable.

## Consent

Written informed consent was obtained from the patient for publication of this case report and any accompanying images. A copy of the written consent is available for review by the Editor-in-Chief of this journal.

## Authors’ information

TS is an assistant doctor, FK is a medical specialist for Otorhinolaryngology, and HR is a medical specialist for Otorhinolaryngology and Head of Department, all within the Department of Otorhinolaryngology, Head and Neck Surgery, Medical University Innsbruck, Austria. SR is an assistant doctor within the Department of Pathology, Medical University Innsbruck, Austria.
